# Supplementary figures and images for: Prenatal and Peripartum Exposure to Antibiotics and Cesarean Section Delivery Are Associated with Differences in Diversity and Composition of the Infant Meconium Microbiome
Source: Microorganisms. 2020 Jan 27;8(2):179. doi: 10.3390/microorganisms8020179 (PMC7074690; doi:10.3390/microorganisms8020179)

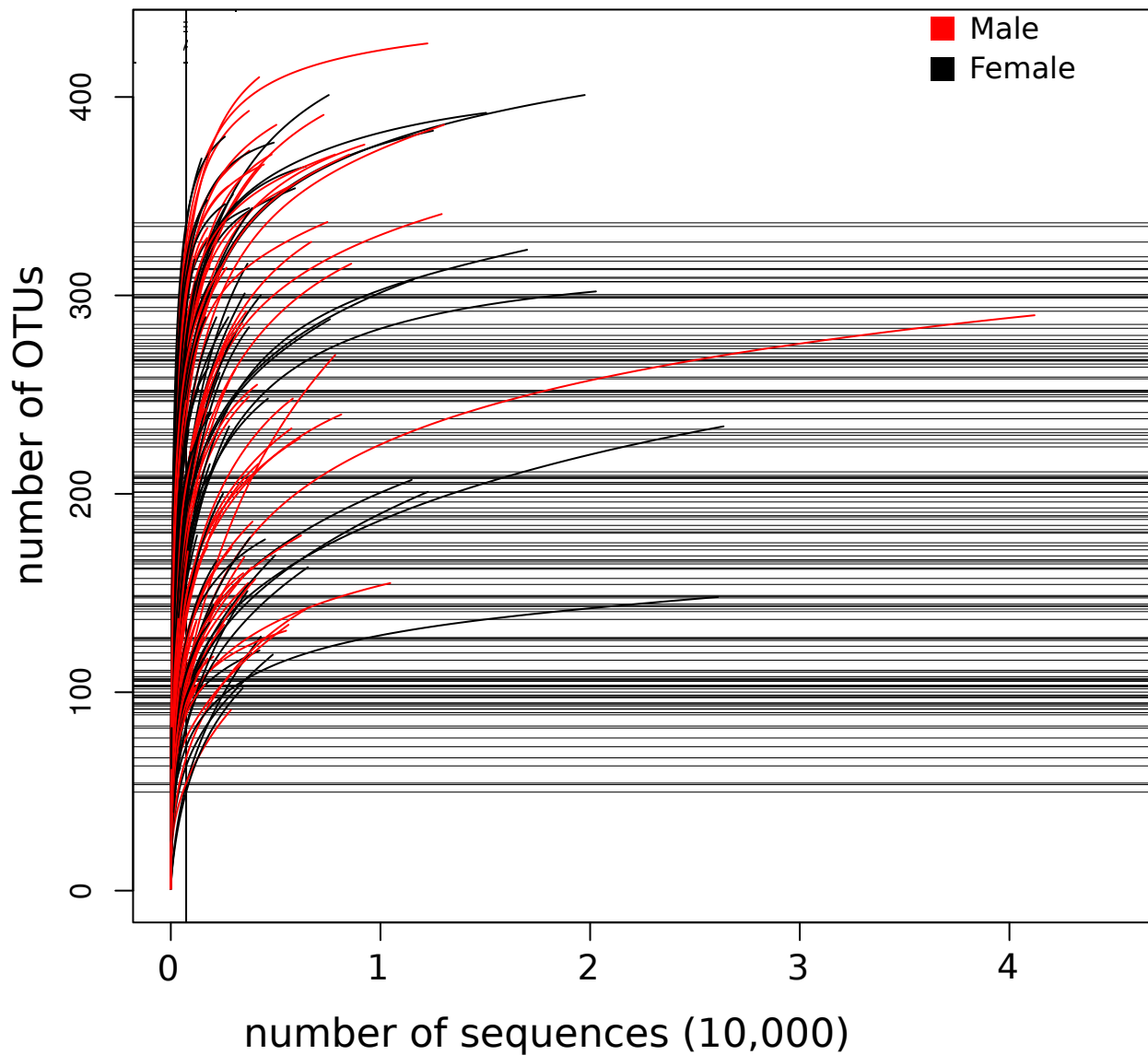

Supplement: Supplementary file 1 [file microorganisms-08-00179-s001.zip › microorganisms-640652-supplementary.pdf]
